# Efgartigimod treatment in patients with anti-MuSK-positive myasthenia gravis in exacerbation

**DOI:** 10.3389/fneur.2024.1486659

**Published:** 2024-11-19

**Authors:** Fangyi Shi, Jiaxin Chen, Li Feng, Rong Lai, Hongyan Zhou, Xunsha Sun, Cunzhou Shen, Jiezhen Feng, Huiyu Feng, Haiyan Wang

**Affiliations:** ^1^Department of Neurology, The First Affiliated Hospital, Sun Yat-sen University, Guangzhou, China; ^2^Guangdong Provincial Key Laboratory of Diagnosis and Treatment of Major Neurological Diseases, Guangzhou, China; ^3^National Key Clinical Department and Key Discipline of Neurology, Guangzhou, China

**Keywords:** myasthenia gravis, MuSK antibodies, efgartigimod, myasthenic crisis, exacerbation

## Abstract

**Background:**

The prevalence of patients positive for muscle-specific kinase antibody (hereafter, MuSK-Ab) accounts for 5–8% of all myasthenia gravis (MG) cases. Currently, efgartigimod has shown good therapeutic effects in MUSK-Ab-positive MG patients in a phase III clinical trial. However, phase III clinical trials tend to exclude MG patients in exacerbation, and there are only few real-world studies on the efficacy of efgartigimod in MuSK-Ab-positive myasthenic crisis (MC) patients. This retrospective, real-world study aimed to explore the efficacy of efgartigimod in MuSK-Ab-positive MG with exacerbation.

**Methods:**

We reviewed the clinical data of four MuSK-Ab-positive patients with exacerbation of MG who received efgartigimod at the First Affiliated Hospital of Sun Yat-sen University, including two patients with MC. All patients were admitted between September 2023 and May 2024. Most patients are simultaneously undergoing rituximab treatment.

**Results:**

Each patient completed one cycle of efgartigimod. After the first administration, four patients showed a clinically meaningful decrease in the Myasthenia Gravis Activities of Daily Living (MG-ADL) score (a reduction of more than 4 points compared to baseline), and all patients showed a decrease in IgG levels after one cycle of efgartigimod. Regarding safety, none of the patients experienced any obvious adverse effects. At the final follow-up, all patients achieved the minimal symptom expression status (an MG-ADL score of 0 or 1) following the first administration of efgartigimod for 8.75 ± 5.56 weeks. This article presents a case involving a patient who exhibited prompt alleviation of symptoms following the administration of a high dose of efgartigimod (20 mg/kg, given on days 1 and 5), without the use of any other fast-acting treatment.

**Conclusion:**

This retrospective real-world study demonstrates the effectiveness and safety of efgartigimod in these four MuSK-Ab-positive, female Asian patients with exacerbation of MG, as well as in patients experiencing MC. It is important to note that efgartigimod should not be viewed as a substitute for foundational immunotherapy; rather, it is intended as a rescue treatment during exacerbations and as an adjunctive therapy in the context of long-term immunotherapy. This non-invasive approach has the potential to become another treatment option for MuSK-Ab-positive MG patients.

## 1 Introduction

Myasthenia gravis (MG) is an antibody-mediated autoimmune disease characterized by fluctuating muscle weakness and fatigue, which can affect the skeletal muscles throughout the body (1, 2). Among all patients with MG, approximately 5–8% are muscle-specific kinase antibody (MuSK-Ab)-positive (3–5). MuSK-Ab-positive patients with MG primarily exhibit symptoms affecting the bulbar, respiratory, and neck muscles. The initial symptoms can include dysarthria, dysphagia, dyspnea, and head drop (5–7). Patients also tend to develop a myasthenic crisis (MC) (5). Nearly 40% MuSK-Ab-positive patients with MG will experience MC (8), and those who are MuSK-Ab-positive and experience MC tend to have a longer tracheal intubation time, longer stay in the intensive care unit (ICU), and longer overall hospitalization time (9).

MuSK-Ab belongs to the IgG4 type that are unable to activate the complement system or mediate AChR receptor internalization; as a result, patients shown poor responsiveness to intravenous immunoglobulin (IVIG) (10). The observed clinical symptoms in patients with MG and MuSK-Ab positivity are closely linked to antibody titers (3). Therefore, reducing antibody titers in the serum can help alleviate the clinical symptoms.

Efgartigimod is a human IgG1 Fc fragment that competitively binds to the neonatal Fc receptor, displaces pathogenic antibodies, and inhibits IgG recycling (11). The efficacy of efgartigimod in non-exacerbation MuSK-Ab-positive patients with MG has been demonstrated in a phase III clinical trial, with all three patients showing positive treatment outcomes as responders on the Myasthenia Gravis Activities of Daily Living (MG-ADL) scale. Notably, phase III clinical trials tend to exclude MG patients in the MGFA V stage, and there are only few real-world studies on the efficacy of efgartigimod in MuSK-Ab-positive patients with exacerbation. Herein, we report the clinical details of four patients with MuSK-Ab-positive MG with exacerbation who were treated with efgartigimod and provide an evaluation of its efficacy. Two of these four patients experienced MC.

## 2 Materials and methods

### 2.1 Ethics approval and consent to participate

All procedures with human participants’ involvement were following the ethical standards of the institutional and/or national research committee and with the 1964 Helsinki Declaration and its later amendments or comparable ethical standards. This is an observational study, and the local ethics committee for clinical research has confirmed that no ethical approval is required.

Before administration of the efgartigimod, we clearly informed the efficacy and AEs of it and fully explained the purpose and content of this study. Moreover, as this is a retrospective study with no additional interventions, the requirement for written informed consent was waived.

All patients indicated agreement for publication. No personal information of the participants has been disclosed in this manuscript.

### 2.2 Patients

Four myasthenia gravis patients with MuSK-Ab positive MG, acute exacerbation, and IgG levels >6 g/L undergoing treatment at the First Affiliated Hospital of Sun Yat-sen University between September 2023 and May 2024 were included. Two were outpatients, and the remaining two inpatients were in MC. Among the outpatients, one exhibited a rapid deterioration of symptoms shortly after catching a cold. The outpatient assessment showed an MG-ADL score of 13 and Myasthenia Gravis Foundation of America (MGFA) class IVB, which can be categorized as impending MC (12): rapid clinical worsening of MG that, in the opinion of the treating physician, could lead to crisis in the short term (from days to weeks). The other outpatient also experienced exacerbation after catching a cold, with an MG-ADL score of 5, mainly presenting symptoms on the bulbar and ocular muscles. Patients who have fulfilled the criteria of CSR (Complete Stable Remission), PR (Pharmacologic Remission), or MM (Minimal Manifestations) but subsequently developed clinical findings greater than permitted by these criteria were said to be in the acute exacerbation period (13). Both MC cases met the international definition of manifested MC: worsening of myasthenic weakness requiring intubation or non-invasive ventilation to avoid intubation, except when these measures are applied in routine postoperative management (12). All patients had received at least one cycle of efgartigimod and had their MG-ADL scores recorded before and after treatment. Patients’ information included antibody status, history of thymoma and thymectomy, and MG-specific treatment at the start of efgartigimod therapy.

### 2.3 Treatment

**Figure 1:**
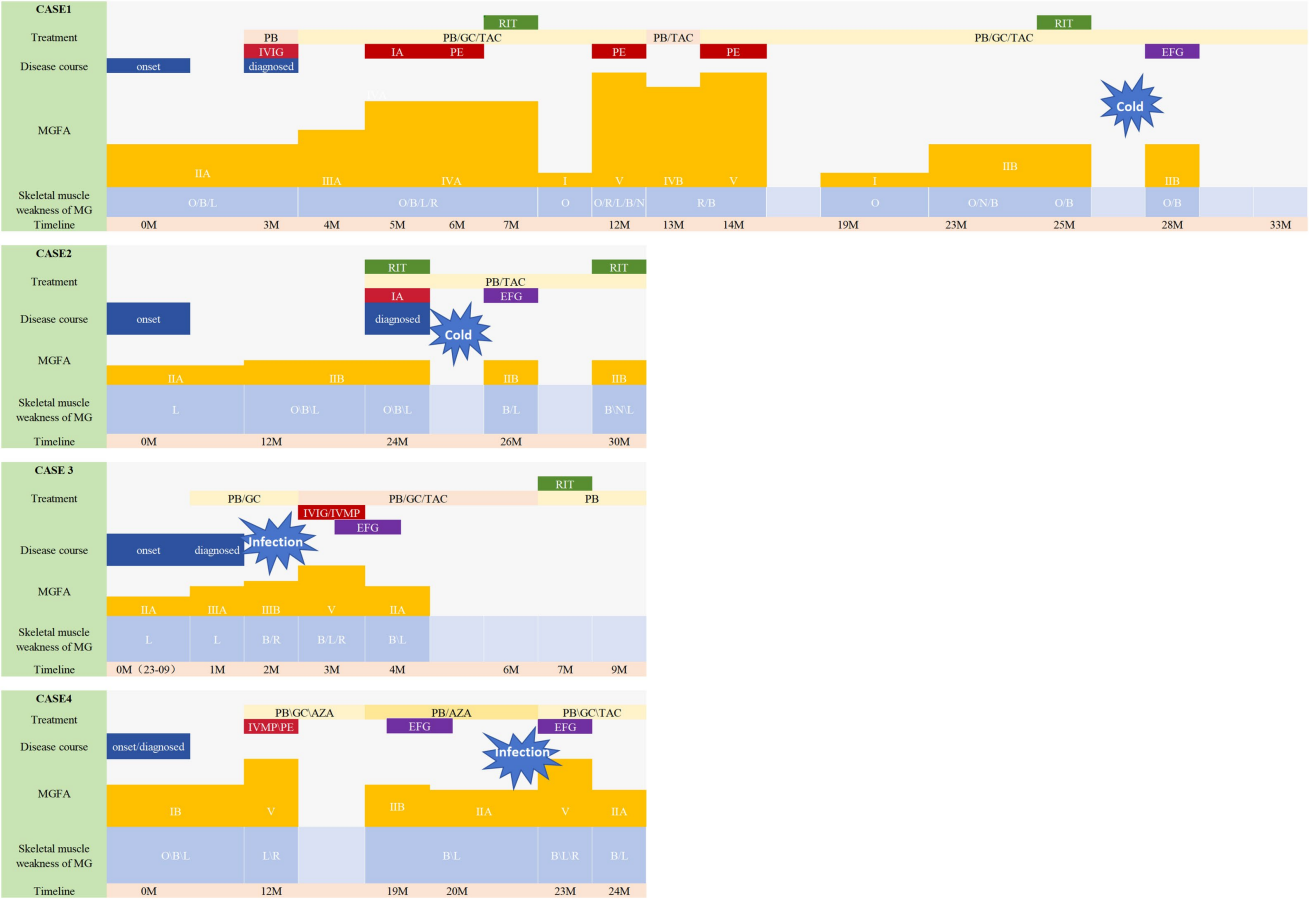
Disease course of the patients. This figure illustrates the progression of the illness and the Myasthenia Gravis Foundation of America (MGFA) classification, along with the specific muscle groups impacted in four patients. Changes in the treatment are also shown along the trajectory. All patients experienced a worsening of symptoms. Case 1 and 2 had been undergoing consistent rituximab therapy prior to efgartigimod administration; however, they exhibited fluctuating symptoms during the course of rituximab treatment. They both were symptom-free for 3–4 months (thus the MGFA classification was not shown), but quickly developed dysarthria and dysphagia after having a cold (MGFA IIb). Notably, their symptoms demonstrated significant improvement following the initiation of efgartigimod therapy. Case 4 had previously undergone a regimen of four weekly injections at other hospital prior to this exacerbation, which resulted in symptom alleviation and a reduction in MGFA classification from IIb to IIa, a status that was sustained for 3 months. They also saw a deterioration in symptoms from MGFA IIA/IIIB to V, indicating a MC. Patient 3 received efgartigimod treatment subsequent to the ineffectiveness of IVMP and IVIG therapies. Similarly, Patient 4 exhibited rapid symptom alleviation following efgartigimod administration, without the need for additional fast-acting treatment. Following the stabilization of his symptoms, Patient 4 is scheduled to resume regular rituximab therapy. MG, myasthenia gravis; MGFA, Myasthenia Gravis Foundation of America; GC, prednisone/methylprednisolone; PB, pyridostigmine; Tac, tacrolimus; RIX, rituximab; EFG, efgartigimod; AZA, azathioprine; B, bulbar muscles; N, neck muscles; R, respiratory muscles; L, limb muscles; PE, pulmonary embolism; PMV, prolapse of mitral valve; T2DM, diabetes mellitus type 2; HP, hypertension; IVIG, intravenous immunoglobulin; PE, plasma exchange; IVMP, intravenous high-dose methylprednisolone.

Three patients received 10 mg/kg efgartigimod administered as four infusions per cycle (one infusion per week), while the remaining patient received 20 mg/kg efgartigimod on day 1 and day 5. Prior to starting efgartigimod, most patients were taking corticosteroids and/or steroid-sparing therapies (including receiving rituximab every 6 months). Medication dosages were gradually tapered or kept constant throughout the treatment process depending on the primary disease course (Table 1). The disease course of the patients is presented in Figure 1.

**Table 1:** Demographics and baseline clinical characteristics of four patients.

	Age at diagnosis	Sex	MG duration	Underlying disease	MGFA	Time of MC onset	Treatment before efgartigimod					Efgartigimod regimen in exacerbation
							ACEIs	Steroids	Immunosuppressive therapies	IVIG	PE	
Case 1	63	Female	2 years	T2DM, HP	IIB	3	Pyd 60 mg tid	CS 5 mg qd	Tac 3 mg qn, Rituximab Q6M			10 mg/kg of intravenous efgartigimod per week
Case 2	42	Female	2 years	NO	IVA	0	Pyd 60 mg qid	CS 12 mg qd	Tac 3 mg qn, Rituximab Q6M			10 mg/kg of intravenous efgartigimod per week
Case 3	51	Female	3 months	NO	V	2	Pyd 60 mg q8h	IVMP–CS 40 mg qd	Tac 3 mg qn, Rituximab Q6M	Yes		10 mg/kg of intravenous efgartigimod per week
Case4	43	Female	2 years	Old pulmonary tuberculosis	V	2	Pyd 60 mg q8h		AZA 50 mg b.i.d			20 mg/kg of intravenous efgartigimod on days 1 and 5

MG, myasthenia gravis; MC, myasthenia crisis; MuSK-Ab, muscle-specific tyrosine kinase antibodies; PE, pulmonary embolism; PMV, prolapse of mitral valve; T2DM, diabetes mellitus type 2; HP, hypertension; IVIG, intravenous immunoglobulin; PE, plasma exchange; IVMP, intravenous high-dose methylprednisolone; GC, prednisone/methylprednisolone; PB, pyridostigmine; Tac, tacrolimus; AZA, azathioprine.

### 2.4 Evaluation of clinical efficacy

The MG-ADL scale was utilized in this study to assess clinical efficacy. A clinically meaningful improvement in the MG-ADL score was defined as a ≥ 4-point reduction after one cycle of efgartigimod administration compared with baseline. Minimal symptom expression (MSE) is defined as an MG-ADL score of 0 or 1 (14–16).

The reduction in patients’ IgG levels was also included to analyze the efficacy of efgartigimod. The concentration of total IgG in plasma was measured by an immunoturbidimetric assay.

### 2.5 Evaluation of safety

All adverse events (AEs) during and after treatment with efgartigimod were reported. The white blood cell count and serum albumin were measured at the initial administration of efgartigimod and at the last follow-up.

## 3 Results

### 3.1 Clinicodemographic characteristics of patients

The mean age of the patients was 49.75 ± 9.71 years, and the mean duration of MG was 1.56 ± 0.88 years. All four patients were female. Chest computed tomography did not reveal thymomas in any patient. Case 1 and Case 2 were in MC and received efgartigimod during hospitalization; the remaining two patients were outpatients (Table 1).

### 3.2 Clinical effectiveness of efgartigimod for the treatment of MG

#### 3.2.1 MG-ADL scores

**Figure 2:**
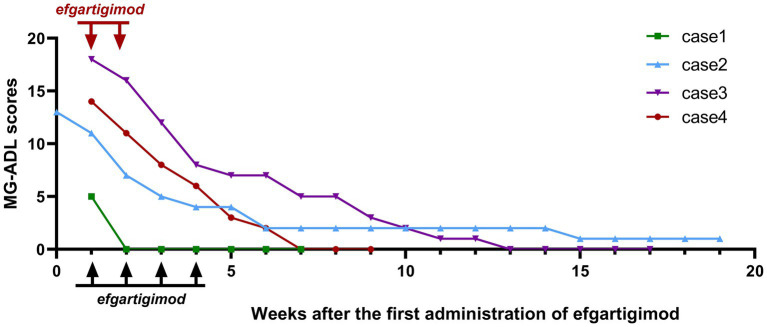
Changes in Myasthenia Gravis Activities of Daily Living (MG-ADL) scores for patients. The black arrows represent the four doses of efgartigimod at 10 mg/kg once a week, while the red arrows represent the two doses (case 4) at 20 mg/kg on day 1 and 5.

The MG-ADL scores of all the four patients were assessed (Figure 2). All patients experienced a clinically meaningful improvement of their symptoms, as evidenced by a ≥ 4 point decrease in the MG-ADL score after one cycle of medication, compared with baseline. The MG-ADL score reduced from 12.00 ± 5.48 at the baseline to 5.75 ± 4.79 after one cycle of efgartigimod.

The follow-up duration for the four patients was 13.00 ± 5.89 weeks. At the final follow-up, four patients ultimately reached the MSE state at 8.75 ± 5.56 weeks after receiving the first administration of efgartigimod. In Case 1, there was a significant improvement in symptoms after the first week of medication, and the MG-ADL score dropped to 0, reaching the MSE state.

#### 3.2.2 IgG

**Figure 3:**
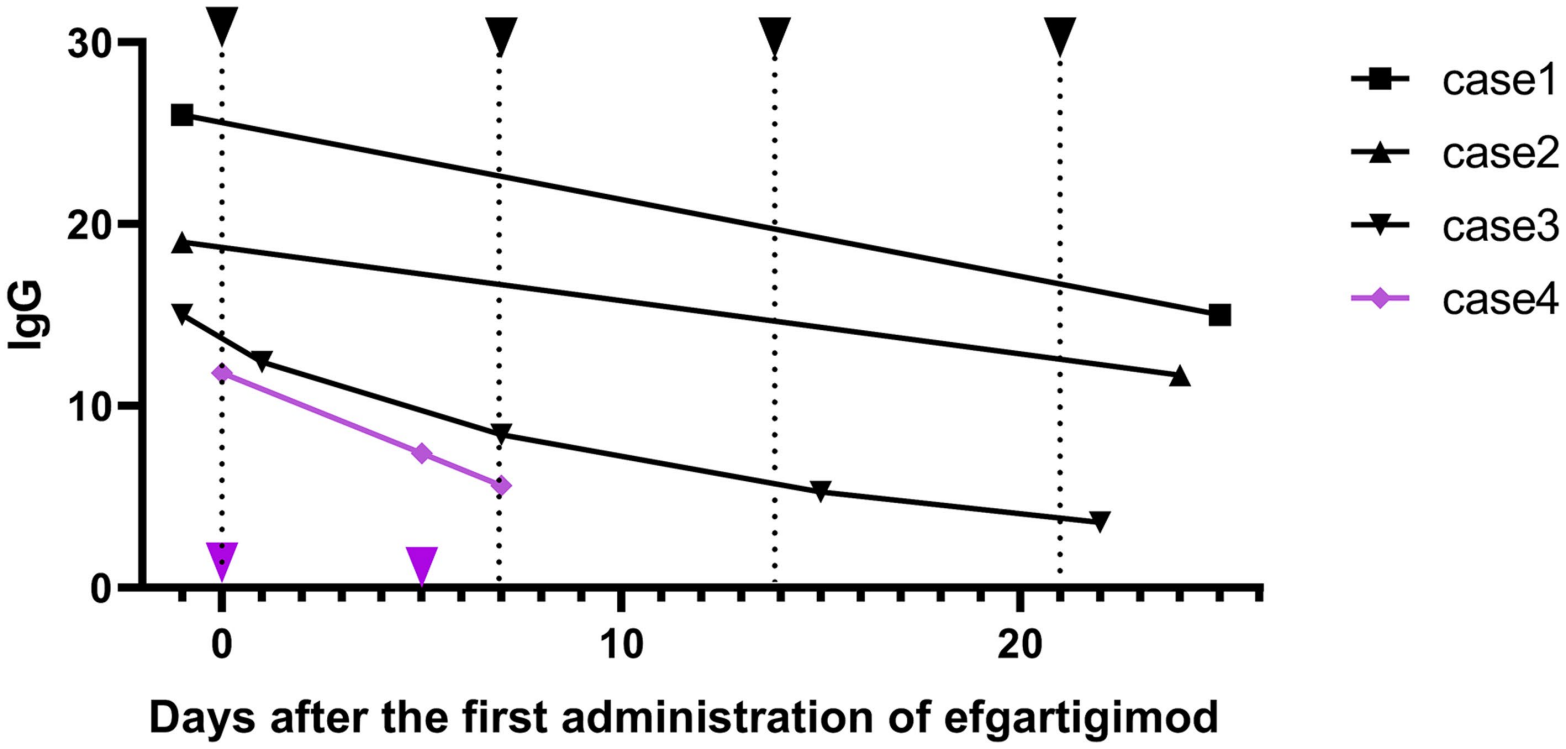
Changes in the immunoglobulin G (IgG) levels before and after one cycle of efgartigimod treatment for patients. The black arrows represent four doses of efgartigimod at 10 mg/kg once a week, while the purple arrows represent one patient (Case 4) receiving efgartigimod at 20 mg/kg on day 1 and 5.

A change in serum IgG levels before and after one cycle of medication was observed in all patients (Figure 3). IgG levels decreased from 17.95 ± 6.12 g/L to 9.42 ± 4.98 g/L, by 52.31 ± 16.9% (42.31, 38.42, 76.07, 52.45%, respectively).

### 3.3 Safety profile of efgartigimod for the treatment of MG

None of the patients experienced any infusion-related reaction events or hypalbuminemia, but Case 4 complained of headache which was tolerable.

## 4 Discussion

This article reports on four patients with MuSK-Ab-positive MG with exacerbation who were treated with efgartigimod. All four patients completed one cycle of treatment, and responded well to efgartigimod, as indicated by a clinically meaningful decrease in the MG-ADL score or a decrease in serum IgG.

All patients in this study received efgartigimod during exacerbation. Two outpatients experienced exacerbation of a cold with the regular use of rituximab. Following efgartigimod treatment, their symptoms improved rapidly. The symptoms of Case 1 disappeared immediately after the first injection and remained stable until the subsequent rituximab treatment. Case 2 successfully reached a stable state after one cycle of efgartigimod treatment before the subsequent rituximab treatment as displayed in Figure 1. Unfortunately, because of insurance-related issues, both patients chose to receive rituximab treatment instead of continuing regular efgartigimod injections.

Case 4 received efgartigimod directly without undergoing any other fast-acting treatments, such as intravenous high-dose methylprednisolone (IVMP), IVIG, and plasma exchange (PE). Both Cases 3 and 4 were in MC and were successfully withdrawn from mechanical ventilation (MV) after efgartigimod treatment (Case 3 was withdrawn on day 4 subsequent to the administration of the second dose of efgartigimod and Case 4, on day 6 after receiving a cycle of efgartigimod) with ICU stay of 22 days and 25 days, respectively, and MV times of 20 days and 19 days, respectively. The length of ICU stays and MV time for these two patients did not show significant differences compared to a large data retrospective study. A median MV duration of 12 days (range: 1–219 days) and median ICU stay of 16 days (17).

Safety was acceptable: one patient experienced tolerable headaches, while another patient experienced changes in laboratory test indices; however, these reactions did not lead to discontinuation of the drug, and all four patients were concurrently taking oral or intravenous steroids, making it challenging to rule out the possibility of steroids and concurrent lung infections as the cause of leukocytosis. At present, there is no verified research to establish efgartigimod’s efficacy in patients with MC. However, given its ease of administration and reduced adverse events, efgartigimod could potentially become a treatment alternative for those with MC.

Treatment alternatives for individuals with MuSK ab-positive MG are relatively scarce, and the clinical outcomes are frequently unsatisfactory. Many patients with MG who test positive for MuSK-Ab typically show limited improvement with various immunosuppressants, leading to low remission rates following such treatments. Rituximab, a chimeric anti-CD20 monoclonal antibody known for B-cell depletion, has shown encouraging results in treating MuSK-Ab-positive MG patients according to various clinical observations, and is even reported to help reduce the use of steroids (12, 18–27). The efficacy and safety of rituximab for MuSK-Ab-positive patients with MG have not been clinically validated by randomized controlled studies, but some retrospective studies have confirmed that rituximab is more effective for MUSK-Ab-positive patients, with approximately 70–80% patients showing response to it, even surpassing patients with AChR antibodies (19–27). Current guidelines recommend early administration of this medication when the standard initial treatment fails to result in prompt rapid remission (12). However, the onset of rituximab’s effects is relatively slow, so it is not suitable for use in exacerbations to rapidly control symptoms.

In this article, Cases 1, 2, and 3 were administered regular infusions of rituximab as maintenance therapy alongside with efgartigimod to control exacerbation symptoms. This choice was based on the pharmacokinetics of efgartigimod, which has a half-life of 80–120 h and is fully metabolized after five half-lives (11). Consequently, we recommend initiating the subsequent rituximab infusions 2–4 weeks following efgartigimod administration to avoid rapid rituximab clearance and maintain therapeutic efficacy. Owing to its ability to enhance the breakdown of IgG immunoglobulins in the body, efgartigimod may potentially influence the metabolism of rituximab, which is classified under the IgG1 subclass and has a median half-life of approximately 22 days (range: 6.1–52 days) (27). This interaction could lead to an accelerated clearance rate of rituximab from previous treatment cycles. Therefore, administering efgartigimod to control acute exacerbations may potentially impact the long-term efficacy of rituximab. However, in our review, we found that two patients (Case 1 and Case 2) did not experience fluctuations in their condition before receiving the next course of rituximab. Thus, we thought that efgartigimod can be used as a rescue treatment during exacerbations to regular rituximab treatment.

Determining the most suitable medication for managing acute exacerbation poses a significant challenge. The decision-making process involves weighing the options of alternative fast-acting treatments or direct administration of efgartigimod. A thorough evaluation should be conducted, taking into account the patient’s clinical condition, drug accessibility, convenience, and other relevant factors. With respect to Case 4, the decision to administer high-dose efgartigimod for prompt shock treatment was based on several considerations, including good respond to patient’s previous use of efgartigimod and its invasive administration method compared to PE. Our clinical experience has shown that high-dose efgartigimod ensures proper dosage while reducing administration time. The decision to utilize a higher dosage and an alternative administration schedule—specifically, the intravenous delivery of 20 mg/kg of efgartigimod on days one and five—derives from the treatment protocol established in the ongoing Phase II clinical trial for Guillain-Barré syndrome (GBS). Findings from the pharmacodynamics study conducted during the Phase I clinical trial of efgartigimod demonstrated that dosages ranging from 10 to 25 mg/kg, administered every 4 to 7 days, did not result in drug accumulation and were deemed safe. Furthermore, we posit that the management approach for patients experiencing MC and classified as IVB differs from that for those IIA-IVA, necessitating short-term, rapidly effective treatment regimens akin to IVIG or PE. Using standard treatment regimens (10 mg/kg once weekly for a total of 4 doses) for patients in exacerbation or MC is may not suitable. The treatment regimen during this phase should be tailed, focusing on the rapid elimination of pathogenic antibodies. This new dosing regimen compresses the original 4-week treatment to 5 days, and the single dose was doubled while maintaining the total dose unchanged. This dosing approach is expected to decrease the treatment duration and facilitate the recovery of patients in exacerbation. Consequently, Case 4 underwent high-dose efgartigimod treatment, followed by rituximab infusion, resulting in favorable clinical outcomes and safety.

Evidence from animal models, case reports, and clinical trial outcomes has shown that efgartigimod is capable of reducing MuSK-Ab levels. In mice, the levels of passively transferred IgG4 antibodies decreased on the first day after efgartigimod infusion, with a faster rate of decrease than the control group. At the endpoint, the levels of MuSK-Ab in the efgartigimod group decreased by 8-fold. Additionally, mice in the efgartigimod group exhibited better results regarding neuromuscular performance, body weight, grip strength, the inverted mesh hang test, and repetitive nerve stimulation than the Fc fragment control group (*p* < 0.05) (28). Similarly, the phase III study ADAPT and ADAPT+ trials confirmed the efficacy and safety of efgartigimod in patients who were positive for MuSK-Ab. However, in the phase I clinical trial, efgartigimod was shown to expedite the clearance of IgG4 antibodies, with a reduction in IgG4 antibodies comparable to IgG1–3 (11).

The strength of this study lies in our pioneering use of efgartigimod to treat MuSK-Ab-positive MG with exacerbation. The study also investigated the effectiveness and safety of efgartigimod in two patients experiencing MC. One of the patients underwent a new high-dose efgartigimod treatment regimen, which reduced the treatment duration while maintaining the same overall dosage. This approach successfully relieved the MC in the patient, leading to a stable condition.

The study is primarily limited by the small number of patients and the short follow-up time. These limitations did not allow for controlling the severity of the patients’ conditions and infection status, potentially interfering with the accurate assessment of the efficacy of efgartigimod. Our patients did not have data about the changes in the antibody titers, and the majority of them only underwent one cycle of efgartigimod treatment. In addition, this study was not a randomized controlled trial. This study exclusively gathered data on the MG-ADL assessments of patients with MC in the ICU owing to their sedated condition, potentially resulting in higher Quantitative Myasthenia Gravis (QMG) scores. Presently, there is a lack of a tailored scoring system for MC patients. Clinical studies are needed to confirm the actual effectiveness of efgartigimod in patients experiencing MC.

## 5 Conclusion

This article discusses the initial real-world investigation into efgartigimod’s application for MuSK-Ab-positive MG patients with exacerbation, featuring two instances of MC; efgartigimod displayed effectiveness and a safety profile. Also, efgartigimod may function as a rescue therapy for patients who are routinely treated with rituximab during exacerbation. For further validation of efgartigimod’s performance as a non-invasive and well-tolerated therapy for MuSK-Ab-positive MG, more rigorous evaluation is required to potentially enhance its clinical impact.

## Data Availability

The original contributions presented in the study are included in the article/supplementary material, further inquiries can be directed to the corresponding authors.

## References

[1] Claytor B Cho S Li Y. Myasthenic crisis. Muscle Nerve. (2023) 68:8–19. doi: 10.1002/mus.27832 37114503

[2] Narayanaswami P Sanders DB Wolfe G Benatar M Cea G Evoli A. International consensus guidance for Management of Myasthenia Gravis: 2020 update. Neurology. (2021) 96:114–22. doi: 10.1212/WNL.0000000000011124, PMID: 33144515 PMC7884987

[3] Bartoccioni E Scuderi F Minicuci GM Marino M Ciaraffa F Evoli A. Anti-MuSK antibodies: correlation with myasthenia gravis severity. Neurology. (2006) 67:505–7. doi: 10.1212/01.wnl.0000228225.23349.5d, PMID: 16894117

[4] Hoch W McConville J Helms S Newsom-Davis J Melms A Vincent A. Auto-antibodies to the receptor tyrosine kinase MuSK in patients with myasthenia gravis without acetylcholine receptor antibodies. Nat Med. (2001) 7:365–8. doi: 10.1038/85520, PMID: 11231638

[5] Borges LS Richman DP. Muscle-specific kinase myasthenia gravis. Front Immunol. (2020) 11:707. doi: 10.3389/fimmu.2020.00707, PMID: 32457737 PMC7225350

[6] Gilhus NE Tzartos S Evoli A Palace J Burns TM Verschuuren JJGM. Myasthenia gravis. Nat Rev Dis Primer. (2019) 5:30. doi: 10.1038/s41572-019-0079-y 31048702

[7] Evoli A Tonali PA Padua L Monaco ML Scuderi F Batocchi AP. Clinical correlates with anti-MuSK antibodies in generalized seronegative myasthenia gravis. Brain. (2003) 126:2304–11. doi: 10.1093/brain/awg223, PMID: 12821509

[8] Evoli A Bianchi MR Riso R Minicuci GM Batocchi AP Servidei S. Response to therapy in myasthenia gravis with anti-MuSK antibodies. Ann N Y Acad Sci. (2008) 1132:76–83. doi: 10.1196/annals.1405.012 18567856

[9] König N Stetefeld HR Dohmen C Mergenthaler P Kohler S Schönenberger S. MuSK-antibodies are associated with worse outcome in myasthenic crisis requiring mechanical ventilation. J Neurol. (2021) 268:4824–33. doi: 10.1007/s00415-021-10603-9, PMID: 33970337 PMC8563593

[10] Herbst R. MuSk function during health and disease. Neurosci Lett. (2020) 716:134676. doi: 10.1016/j.neulet.2019.134676 31811897

[11] Ulrichts P Guglietta A Dreier T van Bragt T Hanssens V Hofman E. Neonatal Fc receptor antagonist efgartigimod safely and sustainably reduces IgGs in humans. J Clin Invest. (2018) 128:4372–86. doi: 10.1172/JCI97911, PMID: 30040076 PMC6159959

[12] Sanders DB Wolfe GI Benatar M Evoli A Gilhus NE Illa I. International consensus guidance for management of myasthenia gravis: executive summary. Neurology. (2016) 87:419–25. doi: 10.1212/WNL.0000000000002790, PMID: 27358333 PMC4977114

[13] Jaretzki A 3rd Barohn RJ Ernstoff RM Kaminski HJ Keesey JC Penn AS. Myasthenia gravis: recommendations for clinical research standards. Task force of the medical scientific advisory Board of the Myasthenia Gravis Foundation of America. Neurology. (2000) 55:16–23. doi: 10.1212/WNL.55.1.16, PMID: 10891897

[14] Uzawa A Ozawa Y Yasuda M Onishi Y Akamine H Kuwabara S. Minimal symptom expression achievement over time in generalized myasthenia gravis. Acta Neurol Belg. (2023) 123:979–82. doi: 10.1007/s13760-022-02162-1, PMID: 36592291

[15] Vissing J Jacob S Fujita KP O'Brien F Howard JF. ‘Minimal symptom expression’ in patients with acetylcholine receptor antibody-positive refractory generalized myasthenia gravis treated with eculizumab. J Neurol. (2020) 267:1991–2001. doi: 10.1007/s00415-020-09770-y, PMID: 32189108 PMC7320935

[16] Katyal N Halldorsdottir K Govindarajan R Shieh P Muley S Reyes P. Safety and outcomes with efgartigimod use for acetylcholine receptor-positive generalized myasthenia gravis in clinical practice. Muscle Nerve. (2023) 68:762–6. doi: 10.1002/mus.27974, PMID: 37695277

[17] Neumann B Angstwurm K Mergenthaler P Kohler S Schönenberger S Bösel J. Myasthenic crisis demanding mechanical ventilation: A multicenter analysis of 250 cases. Neurology. (2020) 94:e299–e313. doi: 10.1212/WNL.0000000000008688 31801833

[18] Díaz-Manera J Martínez-Hernández E Querol L Klooster R Rojas-García R Suárez-Calvet X. Long-lasting treatment effect of rituximab in MuSK myasthenia. Neurology. (2012) 78:189–93. doi: 10.1212/WNL.0b013e3182407982, PMID: 22218276

[19] Hehir MK Hobson-Webb LD Benatar M Barnett C Silvestri NJ Howard JF Jr. Rituximab as treatment for anti-MuSK myasthenia gravis: multicenter blinded prospective review. Neurology. (2017) 89:1069–77. doi: 10.1212/WNL.0000000000004341 28801338

[20] Vesperinas-Castro A Cortés-Vicente E. Rituximab treatment in myasthenia gravis. Front Neurol. (2023) 14:1275533. doi: 10.3389/fneur.2023.1275533, PMID: 37849836 PMC10577386

[21] Vakrakou AG Karachaliou E Chroni E Zouvelou V Tzanetakos D Salakou S. Immunotherapies in MuSK-positive myasthenia gravis; an IgG4 antibody-mediated disease. Front Immunol. (2023) 14:1212757. doi: 10.3389/fimmu.2023.1212757, PMID: 37564637 PMC10410455

[22] Keung B Robeson KR DiCapua DB Rosen JB O'Connor KC Goldstein JM. Long-term benefit of rituximab in MuSK autoantibody myasthenia gravis patients: table 1. J Neurol Neurosurg Psychiatry. (2013) 84:1407–9. doi: 10.1136/jnnp-2012-303664, PMID: 23761915

[23] Beecher G Anderson D Siddiqi ZA. Rituximab in refractory myasthenia gravis: extended prospective study results. Muscle Nerve. (2018) 58:452–5. doi: 10.1002/mus.26156, PMID: 29742795

[24] Litchman T Roy B Kumar A Sharma A Njike V Nowak RJ. Differential response to rituximab in anti-AChR and anti-MuSK positive myasthenia gravis patients: a single-center retrospective study. J Neurol Sci. (2020) 411:116690. doi: 10.1016/j.jns.2020.116690, PMID: 32028072

[25] Zhao C Pu M Chen D Shi J Li Z Guo J. Effectiveness and safety of rituximab for refractory myasthenia gravis: a systematic review and single-arm meta-analysis. Front Neurol. (2021) 12:736190. doi: 10.3389/fneur.2021.736190, PMID: 34721267 PMC8548630

[26] Topakian R Zimprich F Iglseder S Embacher N Guger M Stieglbauer K. High efficacy of rituximab for myasthenia gravis: a comprehensive nationwide study in Austria. J Neurol. (2019) 266:699–706. doi: 10.1007/s00415-019-09191-6, PMID: 30649616

[27] Marino M Basile U Spagni G Napodano C Iorio R Gulli F. Long-lasting rituximab-induced reduction of specific—but not Total—IgG4 in MuSK-positive myasthenia gravis. Front Immunol. (2020) 11:613. doi: 10.3389/fimmu.2020.00613, PMID: 32431692 PMC7214629

[28] Huijbers MG Plomp JJ Van Es IE Fillié-Grijpma YE Kamar-Al Majidi S Ulrichts P. Efgartigimod improves muscle weakness in a mouse model for muscle-specific kinase myasthenia gravis. Exp Neurol. (2019) 317:133–43. doi: 10.1016/j.expneurol.2019.03.001, PMID: 30851266

